# Assessment of Agricultural Carbon Emissions and Their Spatiotemporal Changes in China, 1997–2016

**DOI:** 10.3390/ijerph16173105

**Published:** 2019-08-26

**Authors:** Xiuquan Huang, Xiaocang Xu, Qingqing Wang, Lu Zhang, Xin Gao, Linhong Chen

**Affiliations:** 1 Research Center for Economy of Upper Reaches of the Yangtse River/School of Economics, Chongqing Technology and Business University, Chongqing 400067, China; 2 Business School, Hohai University, Nanjing 211100, China; 3 School of Mathematics and Statistics, Chongqing Technology and Business University, Chongqing 400067, China; 4 School of Public Administration, Sichuan University, Chengdu 610065, China

**Keywords:** agricultural carbon emissions, spatiotemporal differentiation, spatial correlation, temporal evolution, carbon sources

## Abstract

Despite achieving remarkable development, China’s agricultural economy has been under severe environmental pressure. Based on previous studies, the present study further considers the sources of agricultural carbon emissions in depth, estimates China’s agricultural carbon emissions from 1997 to 2016, and analyzes the agricultural pollution faced by China and its provinces. The study estimates the amount and intensity of agricultural carbon emissions in China from five carbon sources—agricultural materials, rice planting, soil N_2_O, livestock and poultry farming, and straw burning—and analyzes their spatial and temporal characteristics. The following results were obtained: (1) between 1997 and 2016, the amount of agricultural carbon emissions in China generally increased, while the intensity of agricultural carbon emissions decreased; (2) in the same period, the amount of carbon emissions from each category of carbon source generally increased, with the exception of rice planting; however, the amount of emissions fluctuated; (3) the amount and intensity of carbon emissions varied greatly among provinces; (4) the emissions from different categories of carbon source showed different concentration trends and agglomeration forms; (5) China’s agricultural carbon emissions showed obvious spatial correlation, which overall was high–high agglomeration; however, its carbon emissions gradually weakened, and the spatial agglomeration of agricultural carbon emissions in each province changed between 1997 and 2016.

## 1. Introduction

Although industry is an important source of carbon emissions, agricultural production also releases large amounts of carbon [1,2]. As the largest carbon emitter in the world, China’s agricultural production activities produce a higher proportion of carbon than any other country. Carbon emissions (including CO_2_, CH_4_, N_2_O, HFCs, PFCs, etc.) are often used to estimate total greenhouse gas emissions. Greenhouse gases from agricultural production account for 10–12% of global greenhouse gas emissions, while in China they account for 16–17% [3], and in the United States, 6–7% [4]. China has made a commitment to reduce its carbon dioxide emission intensity (per unit GDP) by 40 to 50% compared with the 2005 level by 2020 [5]. Therefore, China urgently needs to take measures to reduce agricultural carbon emissions (ACEs), improve resource utilization, and develop low-carbon agriculture. As the focus of low-carbon agricultural development is to control carbon emissions in agricultural development, analyzing the ACEs in China’s provinces can help to develop differentiated policies to promote the coordinated development of low-carbon agriculture in various regions [6]. 

In recent years, research on all aspects of ACEs has gradually developed. In this paper, this previous research is divided into several categories. 

The first category is the study of ACEs from a particular perspective. Some scholars have studied the impact of land use on ACEs at various levels, and found that different uses of agricultural land affect agricultural management models and energy use, thus also affecting ACEs [7,8,9]. Other scholars have studied the relationship between ACEs, energy consumption, agricultural management models, and economic growth, and found that there was a positive relationship between energy consumption and economic growth [10,11,12,13,14,15,16,17,18], meaning that large energy consumption generally causes high carbon emissions. Other studies focused on emission reduction mechanisms [19,20,21,22], which has been a popular research area in recent years.

The second research category is the measurement of ACEs and the analysis of their distribution characteristics, which is the basis for in-depth analysis of ACEs. Since it is difficult to improve methods for measuring ACEs, progress in this direction is mostly based on the selection of agricultural carbon emission sources (ACESs). For example, West et al. conducted a detailed study of ACEs, and concluded that ACESs were mainly from agricultural inputs, including fertilizers, agricultural limes, pesticides, agricultural irrigation, and seed cultivation [23]. Johnson et al. argued that ACESs were mainly derived from intestinal fermentation in livestock, manure management, rice growth, and the arbitrary disposal of agricultural waste [24]. Other related studies were more detailed; for example, Tan et al. and Wu and Kaiwen investigated ACESs with respect to four aspects: rice planting, rice fields, input of agricultural material, livestock and poultry farming, and soil management. The indicators were comprehensive; however, the specific carbon emission coefficient was not listed in the studies [25,26].

This study calculated the amount and the intensity of ACEs in China by choosing five categories of ACES: agricultural materials, rice cultivation, N_2_O emissions caused by damage to the soil surface when planting crops, livestock and poultry farming, and crop straw burning. Compared to other research, this research more comprehensively and deeply considered ACESs. Additionally, unlike in other research, the species of rice, crops on the land, and livestock and poultry, and the location of rice, were taken into account when calculating the amount of carbon emissions from rice planting, soil N_2_O, and livestock and poultry farming, since these factors can influence the carbon emissions of those ACESs. Furthermore, this study included agricultural straw combustion as one of the carbon emissions sources, which increased the estimation accuracy for ACEs.

This paper is structured into four main sections. The introduction is followed by the materials and methods section. Section 3 presents the temporal and spatial characteristics of ACEs in China. The discussion and conclusions are presented in Section 4.

## 2. Materials and Methods

### 2.1. Estimation of Agricultural Carbon Emissions (ACEs)

To estimate ACEs in China, it was necessary to develop an agricultural carbon emission estimation system. The study investigated ACEs based on two aspects: the amount of agricultural carbon emissions (AACEs) and the intensity of agricultural carbon emissions (IACEs). Each carbon emission source produces a certain amount of carbon, which can be represented by a corresponding carbon emission coefficient. These coefficients are calculated by relevant professional researchers through experiments. Using carbon emission coefficients, the amount of ACESs can be converted into the AACEs using Equation (1), below, and the IACEs can be obtained by dividing the amount by the total real carbon emissions (based on 1997 levels) from agriculture, forestry, animal husbandry, and fishery.

(1) $$E=\sum E_{i}=\sum Q_{i}\times a_{i}$$

where E represents the amount of carbon emissions from agriculture, $E_{i}$ represents the amount of carbon emission from various types of carbon sources, $Q_{i}$ represents the number of carbon sources, and $a_{i}$ represents the carbon emission coefficient of every carbon source.

After calculating the ACEs for 31 Chinese provinces, the spatial and temporal characteristics of the AACEs and the IACEs were analyzed, mainly using a descriptive analysis method. Additionally, the Moran index was calculated by Stata14.0 (StataCorp LP, College Station, TX, USA) to analyze the spatial correlation of both AACEs and IACEs.

### 2.2. Sample Selection and Data Sources

In China, the natural conditions of agricultural production vary significantly between provinces and regional agricultural development is uneven; therefore, ACEs also vary across the country. Although the ACEs of all provinces should ideally be measured, Hong Kong, Macao, and Taiwan were excluded from this study due to a lack of data availability. In 1997, the city of Chongqing was designated as a municipality directly under the central government, and consequently no independent data are available before that year. The study period was set as 1997–2016, since data was only available up to 2016.

Relevant data were obtained for 31 provinces and were then used to calculate ACEs. The data were obtained from the China Rural Statistical Yearbook, the China Agricultural Yearbook, China Agricultural Statistics, the China Animal Husbandry Yearbook, and the Statistical Yearbook of China.

### 2.3. Sources of ACEs

#### 2.3.1. Carbon Emissions from Agricultural Materials

In previous research, ACEs were mainly estimated based on the use of two major types of agricultural materials in agricultural production activities: the direct input of agricultural materials as production factors, such as fertilizers, pesticides, and plastic sheeting; and energy consumption in agricultural production activities, such as electricity and diesel oil consumption. Following previous research [27,28], this study selected fertilizers, pesticides, plastic sheeting, diesel oil, and irrigation as carbon sources from agricultural materials, and their carbon emission coefficients were taken as 0.8956, 4.9341, 5.18, 0.5927, and 266.48 kg C/kg, respectively.

#### 2.3.2. Carbon Emissions from Rice Cultivation.

China cultivates a huge amount of rice, having the highest rice yield in the world. Accordingly, carbon emissions due to rice cultivation contribute significantly to China’s ACEs, mainly through the production of the greenhouse gas CH_4_. Although the production of other dryland crops also emits CH_4_ and dryland itself can absorb CH_4_, this study ignored the influences of these factors when calculating the AACEs due to their relatively small contribution to ACEs. Both the geographical location and the species of rice were taken into account when calculating carbon emissions from paddy fields, which was the key goal of this study. The coefficients of CH_4_ emissions are shown in Table 1 [29].

#### 2.3.3. N_2_O Emissions Caused by Damage to Soil Surface during Crop Planting

When crops are planted, turning the soil causes CO_2_ and N_2_O in the soil to flow into the air. However, in this study, CO_2_ released by soil turning was ignored in the calculation of carbon emissions due to its small contribution and the absorption of CO_2_ by photosynthesis during crop growth. Following relevant research [30,31], the N_2_O emission coefficients of soil for paddy rice, winter wheat, spring wheat, soybean, corn, and vegetables were taken as 0.24, 2.05, 0.4, 0.77, 2.532, and 4.21 g/m^2^, respectively.

#### 2.3.4. Carbon Emissions from Livestock and Poultry Farming

Greenhouse gases, mainly CH_4_ and N_2_O, are also produced during livestock and poultry farming activities by manure management systems and enteric fermentation. Since the breeding cycles of livestock and poultry are not the same, it was necessary to adjust the amount of feeding. The average life cycles of pigs, rabbits, and poultry were 200 days, 105 days, and 55 days, respectively, and the slaughter rate was greater than 1, which was adjusted according to Equation (2):

(2) $$N_{i}=DL_{i}\times \frac{M_{i}}{365}$$

where $N_{i}$ is the average annual feeding amount for livestock and poultry, $DL_{i}$ is the average life cycle of the animals, and $M_{i}$ is the annual output.

The output rate of the selected livestock and poultry was less than 1, and it was therefore adjusted according to Equation (3):

(3) $$N_{i}=\frac{C_{i,t}+C_{i,t-1}}{2}$$

where $N_{i}$ is the average annual feeding amount, and $C_{i,t}$ and $C_{i,t-1}$ represent the year-end inventory of years t and t−1, respectively.

The 12 most important animals in Chinese agriculture were used for the calculation system, and the respective CH_4_ and N_2_O emission coefficients are listed in Table 2 [28].

The CH_4_ and N_2_O emissions must ultimately be converted to standard carbon emissions. According to the IPCC (2007) [28], in terms of the greenhouse effect, 1 ton of CH_4_ is equivalent to 6.8182 tons of C and 1 ton of N_2_O is equivalent to 81.2727 tons of C. Therefore, the CH_4_ and N_2_O emissions can be converted into equivalent C emissions using Equation (4):

(4) $$C=C_{CH_{4}}+C_{N_{2}O}=\sum CH_{4}\times 6.8182+\sum N_{2}O\times 81.2727.$$

#### 2.3.5. Carbon Emissions from Straw Burning

Straw treatment has always been a concern for scholars. In the context of vigorous green development, the combustion of straw has gradually been reduced in recent years; however, such combustion had been an important source of ACEs for a long time. The six main straw crops—rice, wheat, corn, rapeseed, soybean, and cotton—were selected as the carbon sources for straw burning. Following [32], the carbon emission coefficients of rice, wheat, corn, rapeseed, soybean, and cotton straw were taken as 0.18, 0.16, 0.17, 0.22, 0.15, and 0.13 kg C/kg, respectively.

## 3. Results

### 3.1. Evolution of China’s ACEs

The present study estimated China’s AACEs and IACEs from 1997 to 2016. China’s AACEs totaled 311.9183 million tons and 373.9123 million tons in 1997 and 2016, respectively, growing by 19.8% with an average annual growth rate of 0.959%. China’s IACEs were 0.127 kg/yuan and 0.064 kg/yuan in 1997 and 2016, respectively, falling by 49.6% with an average annual decline of 3.5%. The AACEs increased during 2007–2015 and fluctuated at other times, whereas the IACEs declined every year. Given the gradual development of China’s agricultural sector, the relatively small increase in the AACEs and the continuous decline in IACEs indicate that the government’s low-carbon agricultural plan has been vigorously developed.

In 1997, the carbon emissions from various sources (in millions of tons) were as follows: agricultural materials, 68.6382; soil, 13.7568; rice planting, 66.7494; livestock and poultry farming, 84.2977; and straw burning, 78.5061. The carbon emissions from these five sources accounted for 22, 21.4, 4.4, 27, and 25.2% of China’s total carbon emissions, respectively. Meanwhile, in 2016, the carbon emissions from the same sources (in millions of tons) were as follows: agricultural materials, 106.1024; soil, 20.0672; rice planting, 61.4076; livestock and poultry farming, 101.0428; and straw burning, 85.2924. The carbon emissions from these five sources accounted for 28.4%, 16.4%, 5.4%, 22.8%, and 27% of China’s total carbon emissions, respectively.

#### 3.1.1. Evolutional Characteristics of China’s ACEs

During the sample period, China’s AACEs generally increased. The AACEs was in a state of fluctuation until 2006. The AACEs increased from 311.9183 million tons in 1997 to 342.9614 million tons in 2006, growing about by 10%. The AACEs presented an increasing trend from 2007 to 2015, growing from 331.9729 million tons in 2008 to 379.9784 million tons in 2015, an increase of 13.2%, and a declining trend in 2016, when it fell to 373.9123 million tons. 

**Figure 1:**
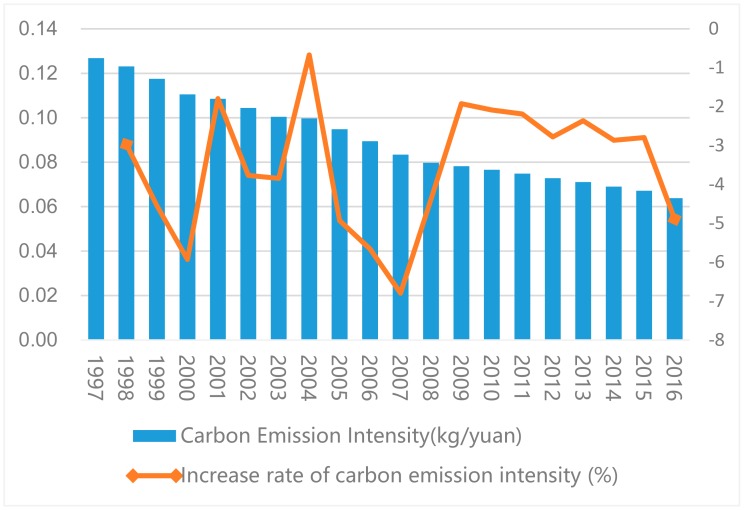
The intensity of agricultural carbon emissions and its growth rate from 1997 to 2016 in China.

Figure 1 shows the IACEs and their growth rate from 1997 to 2016 in China. The intensity decreased every year, falling from 0.127 kg/yuan in 1997 to 0.064 kg/yuan in 2016, representing a total decrease of 49.6% and an average annual decrease of 3.5%. 

The change trends of the intensity and amount of ACEs were strongly consistent over the study period. From 1998 to 2004, the growth rates of ACEs fluctuated, and the inflection points of the growth rates occurred at the same times; however, the ranges of the growth rates were different. From 2004 to 2009, a “V”-shaped growth trend was formed. In 2007, the intensity and amount of ACEs decreased the most, by −6.8% and −3.2%, respectively. After 2008, the decline of the IACEs gradually slowed down; this can be attributed to the gradual increase in production activities after 2008. China’s economy was not significantly influenced by the financial crisis of 2008. From 2009 to 2015, the intensity of ACEs declined steadily, and a slightly larger decline occurred in 2016 when China’s economy declined.

#### 3.1.2. Evolutional Characteristics of the Amount of ACEs by Source

In terms of the total amount of ACEs, the overall carbon emissions from all kinds of sources increased during the study period; the only exception to this was for rice planting, the carbon emissions from which were 8% lower in 2016 than in 1997, with an average annual decline of 0.4% during this period. The amount of carbon emissions from agricultural inputs rose from 68.6382 million tons in 1997 to 106.1024 million tons in 2016, a total increase of 54.6% and an average annual increase of 2.32%. This increase was due to the fact that the growth of China’s agricultural output depended largely on increasing investment in agricultural inputs. Soil N_2_O emissions experienced the second highest increase during the study period, increasing from 13.7568 million tons in 1997 to 20.0672 million tons in 2016, a total increase of 45.87% and an average annual increase of 2%. The amount of carbon emissions from straw burning increased by 28.7% during the study period, with an average annual increase of 1.34%. The smallest change was observed for carbon emissions from livestock and poultry farming, which rose by 1.2% over the study period, with an average annual increase of 0.06%.

**Figure 2:**
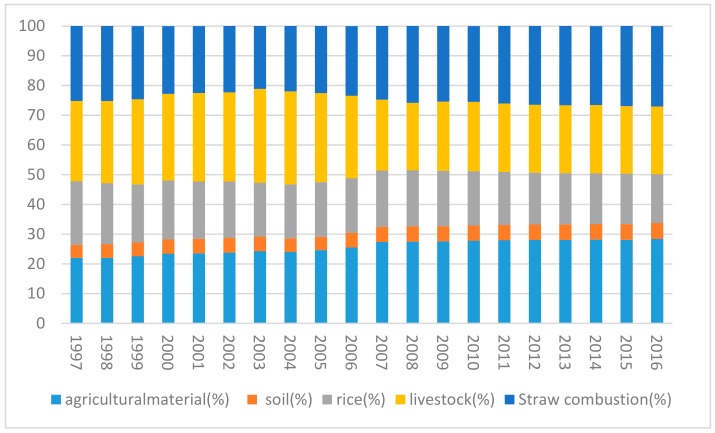
Changes in agricultural carbon emissions in China by source from 1997 to 2016.

The contributions to China’s AACEs from different sources from 1997 to 2016 are shown in Figure 2. The contribution of agricultural inputs to the AACEs gradually increased, from 22% in 1997 to 28.38% in 2016, an increase of about 19%. This increase is related to the increasing dependence of agricultural output on agricultural inputs. The contribution of rice planting to the AACEs declined from 21.39% in 1997 to 16.42% in 2016, and the contribution from livestock and poultry farming decreased from 27.02% in 1997 to 22.81% in 2016. The proportion of ACEs from livestock and poultry farming gradually increased in 1997–2004 and declined in 2004–2016. These changes can be assumed to be mainly due to the adjustment of breeding scale and breed. The proportion of ACEs from soil N_2_O emissions and straw burning respectively increased from 4.41% and 25.17% in 1997 to 5.37% and 27.02% in 2016.

### 3.2. Spatial Variation of China’s ACEs

#### 3.2.1. Spatial Evolutional Characteristics of ACEs

**Figure 3:**
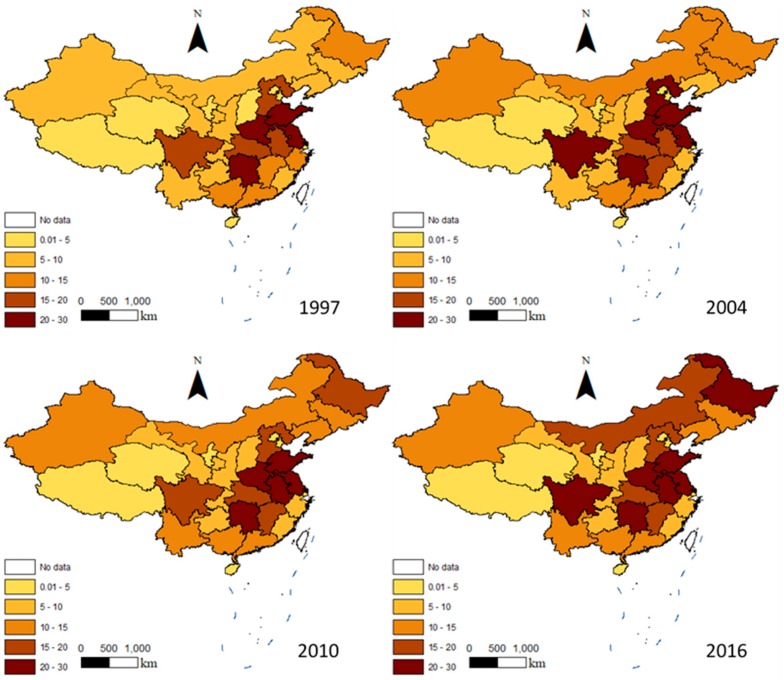
Spatial distribution of the amount of agricultural carbon emissions in China in 1997, 2004, 2010, and 2016 (unit: million tons).

The spatial distribution of China’s AACEs in 1997, 2004, 2010, and 2016 is shown in Figure 3. The AACEs gradually increased between 1997 and 2016, and differed between provinces, with these differences increasing over the study period.

Since agricultural production varies between provinces in China, there was a large difference in the AACEs between the various provinces. Taking the data from 2016 as an example, seven provinces had an AACEs above 20 million tons, namely Henan (29.34127 million tons), Shandong (23.72729 million tons), Heilongjiang (22.67473 million tons), Hunan (22.23557 million tons), Jiangsu (22.1594 million tons), Anhui (21.68624 million tons), and Sichuan (20.34214 million tons). The seven provinces with the lowest ACEs were Beijing (0.53275 million tons), Shanghai (0.98221 million tons), Tianjin (1.16245 million tons), Ningxia (2.4168 million tons), Hainan (2.45597 million tons), Tibet (3.56095 million tons), and Qinghai (3.58645 million tons). The emissions from these seven provinces accounted for 3.9% of the total, while the emissions from the seven highest-emitting provinces accounted for 43%. The emissions from Henan Province, the province with the highest emissions, were 55 times higher than those from Beijing Municipality, showing how unbalanced the AACEs were across China.

The provinces with the highest ACEs were mostly China’s traditional agricultural provinces, with the top 10 being China’s main grain producing areas. China’s major agricultural provinces bear the main responsibility for China’s food security. In these provinces, the main goal of agricultural development is obtaining high grain yields of grain; this means that the production model is relatively simple and the AACEs are relatively high. Achieving lower carbon emissions while ensuring sufficient agricultural production is a challenge that needs to be addressed by the Chinese government.

**Figure 4:**
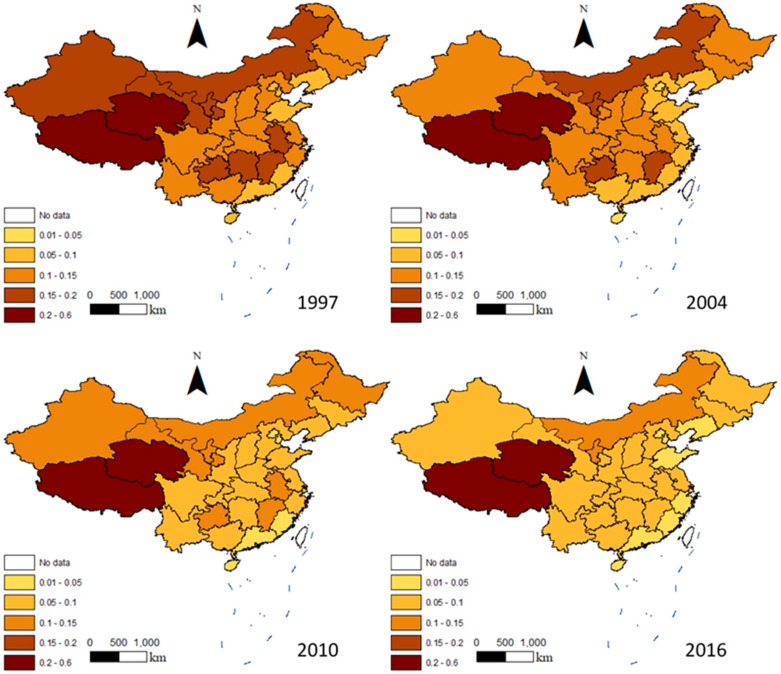
The spatial distribution of the intensity of China’s agricultural carbon emissions in 1997, 2004, 2010, and 2016 (unit: kg/yuan).

Figure 4 shows the spatial distribution of China’s IACEs in 1997, 2004, 2010, and 2016. The IACEs showed an overall downward trend over the study period, with fluctuation being observed in the middle of the study period. However, in some provinces, the IACEs increased, which is consistent with the national trend of IACE evolution.

The spatial distribution of the IACEs was highly uneven, and this unevenness increased over time. In 2016, Tibet’s carbon intensity was the highest among all the provinces, reaching 0.3706 kg/yuan, while Beijing’s was the lowest, reaching 0.0222 kg/yuan. Based on the IACEs, China’s provinces can be divided into three groups: the first group includes the provinces with IACEs higher than 0.1 kg/yuan, that is, Ningxia, Inner Mongolia, Qinghai, and Tibet; the second group includes the provinces with IACEs between 0.05 and 0.1 kg/yuan, namely Hebei, Guangxi, Shaanxi, Jiangsu, Shanghai, Chongqing, Sichuan, Henan, Hubei, Yunnan, Hunan, Guizhou, Jilin, Shaanxi, Anhui, Gansu, Jiangxi, and Heilongjiang; and the third group includes the provinces with IACEs less than 0.05 kg/yuan, namely Beijing, Hainan, Fujian, Tianjin, Guangdong, Zhejiang, Liaoning, and Shandong. 

Most of China’s provinces are in the second group. The first group consists of economically underdeveloped provinces in Western China. Most of the provinces in the third group are in relatively developed areas in Eastern China. On the whole, the IACEs of the provinces in Western China were the highest, followed by provinces in Central China and finally provinces in Eastern China, whose IACEs were the lowest.

#### 3.2.2. Spatial Evolutional Characteristics of ACEs by Source

**Figure 5:**
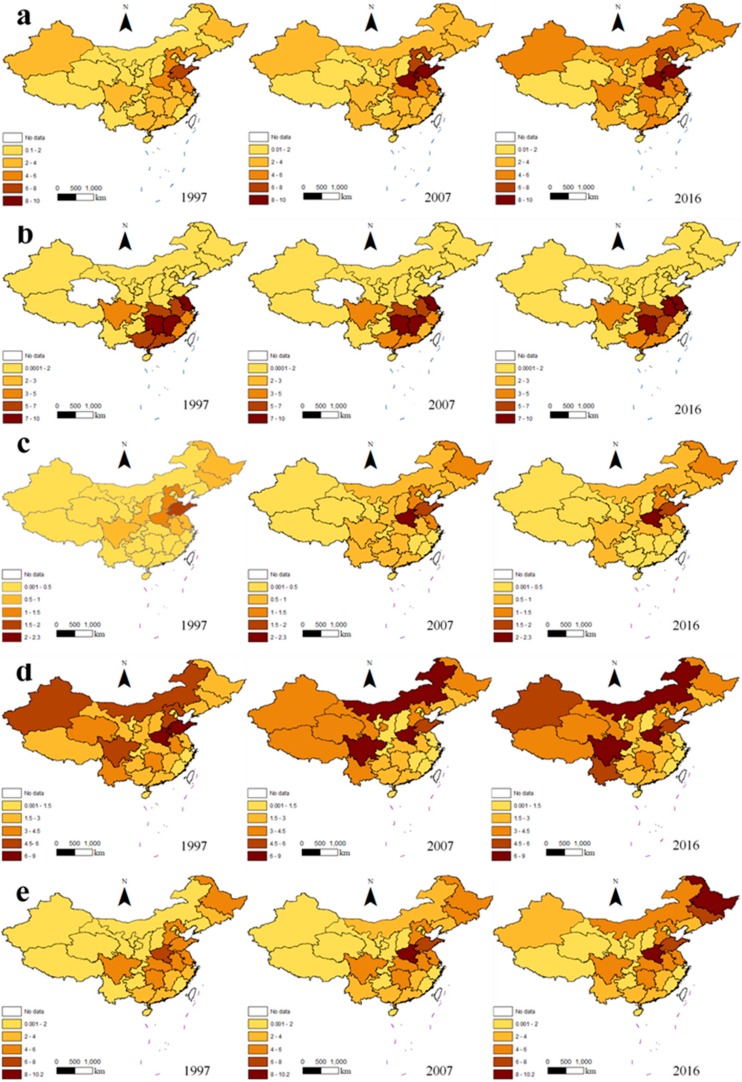
The distribution of carbon emissions from five categories of carbon source in China’s provinces in 1997, 2007, and 2016 (unit: million tons): (**a**) emissions from agricultural materials; (**b**) emissions from rice planting; (**c**) emissions from soil; (**d**) emissions from livestock and poultry farming; (**e**) emissions from straw combustion.

Figure 5 shows the distribution of carbon emissions for the five categories of ACES in China’s provinces in 1997, 2007, and 2016. China has a vast territory, and there are clear regional differences in climatic conditions and resource endowments, and consequently the agricultural production conditions differ in various provinces. Therefore, the agricultural structures of China’s provinces differ, and in turn there is a large difference in the structure of agricultural carbon emissions across the country. 

As shown in Figure 5, over the study period, there was an increase in carbon emissions from agricultural materials, rice planting, soil, livestock and poultry breeding, and straw burning. The carbon emissions from rice planting gradually decreased, except for individual provinces. The AACEs were obviously different in different areas and had an uneven distribution. Additionally, the carbon emissions from different sources showed different concentration trends and agglomeration forms.

Based on the differences in the agglomeration forms of the carbon emissions from different sources, this paper divides the 31 provinces into the following five categories: (1) regions whose carbon emissions were dominantly from agricultural materials. The ACEs in these areas mainly came from agricultural material input in agricultural production activities, while the carbon emissions from other sources were relatively low. As can be seen from Figure 5, this category mainly includes the provinces of Tianjin, Shandong, and Shaanxi; of these, Shandong and Shaanxi were large agricultural producers; (2) regions whose carbon emissions were dominantly from rice planting. These regions include the provinces of Hunan, Jiangxi, Jiangsu, and Anhui. The AACEs in these areas was mainly from rice growth, while the proportion of carbon emissions from agricultural inputs and livestock farming was low; (3) regions dominated by livestock and poultry breeding. Agricultural carbon emissions in these regions—which included the provinces of Tibet, Qinghai, Inner Mongolia, Guizhou, Yunnan, and Sichuan—were mainly due to livestock and poultry rearing. As can be seen from Figure 5, the livestock industry accounted for a large proportion of the ACEs in these areas. The western provinces of Guizhou, Yunnan, and Sichuan are mountainous areas and their economic development is relatively poor. A large number of working animals are needed for agricultural production in the three areas, which contribute significantly to the areas’ carbon emissions; (4) regions dominated by composite carbon sources. In these regions, agricultural carbon emissions were mainly derived from two or more carbon sources. These regions include nine provinces, namely Beijing, Hebei, Shaanxi, Liaoning, Shanghai, Zhejiang, Gansu, Ningxia, and Xinjiang. Some of these areas are located in China’s main grain-producing areas, and some have a relatively balanced development of crop production and animal husbandry; and (5) regions where carbon emissions are spread relatively evenly across the five sources. These regions include the provinces of Fujian, Henan, Hubei, Guangdong, Guangxi, and Hainan. The farming industries in these six provinces were relatively developed, with rice being the dominant food crop and livestock and poultry farming also being relatively well developed.

#### 3.2.3. Analysis of the Spatial Correlation of China’s ACEs

Similar climatic conditions, resource endowments, and agricultural policies had similar effects on agricultural production activities, which led to spatial variability in the distribution of ACEs. Provincial economic units did not exist in isolation, and it was therefore necessary to continue to investigate the interaction effects between agricultural carbon emission units and to investigate the spatial correlation characteristics.

In this study, the widely used Moran index was used to analyze the spatial correlation of China’s ACEs. The Moran index comprises the global Moran index and the local Moran index. The former is used to explore the agglomeration trend of the analytical variable in the whole study space, and the latter is used to analyze the spatial heterogeneity. The stata14.0 software was used to calculate the global Moran index of China’s AACEs and IACEs from 1997 to 2016, and two different weight matrices were used in order to ensure the reliability of the conclusion: the first-order adjacent weight matrix (W_q_) and the geographic distance matrix (W_d_). The results are shown in Table 3. During 1997–2016, the Moran indexes of the AACEs and IACEs were both positive and passed the significance test. Positive values of the Moran index represent the convergence of the high value of the variable (i.e., high–high agglomeration). During the study period, the amount and intensity of ACEs in China showed a positive spatial spillover effect. The greater the value of the Moran index, the stronger the correlation, and vice versa. The Moran indexes of the AACEs and IACEs generally fluctuated during the study period, indicating that the spatial correlation also fluctuated.

**Figure 6:**
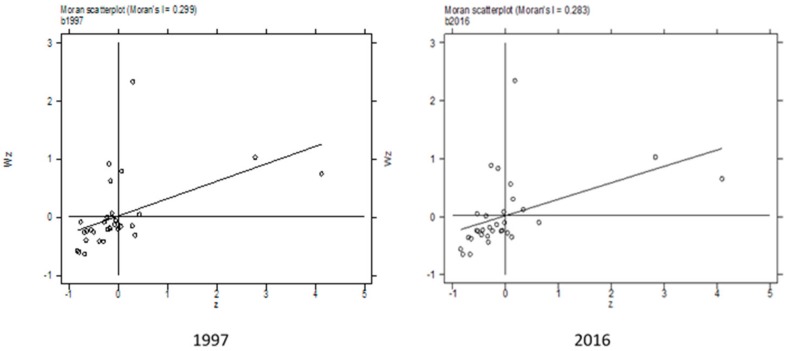
Moran scatter plots of the intensity of agricultural carbon emissions in China in 1997 and 2016.

The global Moran index cannot reflect the spatial correlation of China’s internal provinces. Therefore, we constructed Moran scatter plots of China’s IACEs to analyze the spatial agglomeration of the IACEs in China’s provinces, as shown in Figure 6. The first, second, third, and fourth quadrants of the Moran scatter plots represent high–high type agglomeration areas, low–high type agglomeration areas, low–low type agglomeration areas, and high–low type agglomeration areas, respectively.

In the Moran scatter plot of the IACEs in 1997, five provinces (Tibet, Gansu, Qinghai, Ningxia, and Xinjiang) were located in the high–high agglomeration area, meaning that they had high IACEs and were surrounded by provinces with high IACEs. Three provinces (Heilongjiang, Sichuan, and Shaanxi) were located in the low–high agglomeration area, meaning that these provinces had low IACEs and were surrounded by provinces with high IACEs. Twenty provinces (Beijing, Tianjin, Hebei, Liaoning, Jilin, Shanghai, Jiangsu, Zhejiang, Anhui, Fujian, Shandong, Henan, Hubei, Hunan, Guangdong, Guangxi, Hainan, Chongqing, Shaanxi, and Yunnan) were located in the low–low agglomeration area, meaning that these provinces had low IACEs and were surrounded by provinces with low IACEs. The provinces of Guizhou, Inner Mongolia, and Jiangxi were located in the high–low agglomeration area, meaning that these provinces had high IACEs and were surrounded by provinces with low IACEs. 

In the Moran scatter plot for 2016, compared with the plot for 1997, the high–high agglomeration area now included Heilongjiang Province, with a total of six provinces being located in this area; the low–high agglomeration area no longer included Heilongjiang Province, although it now included the provinces of Liaoning, Jilin, and Shaanxi, making a total of five provinces. The low–low agglomeration area no longer included the provinces of Liaoning, Jilin, Tianjin, or Shaanxi, although it now included Guizhou Province, making a total of 17 provinces. The high–low agglomeration area no longer included Guizhou Province, although it now included Tianjin Province, leaving the number of provinces in this area unchanged. That is, in the 2016 Moran scatter plot, the high–high and low–high agglomeration areas included more provinces than in the 1997 scatter plot, the low–low agglomeration area contained fewer provinces, and the high–low agglomeration area contained the same number of provinces.

## 4. Discussion

Previous studies did not consider enough carbon emission sources when estimating ACEs. Some early studies only selected farming materials as the carbon source, while some later studies additionally selected rice planting as a source, albeit without considering that the species of rice and the location of the field impact the carbon emission coefficients. Additionally, when choosing soil N_2_O as one of the sources, some researchers did not take into account the fact that soil N_2_O emission coefficients vary with the type of crop. In recent years, there have been some relatively comprehensive studies of ACEs; however, these still omitted some important carbon sources. Hence, this study attempted to make some improvements upon previous studies, focusing on two aspects. 

Firstly, this study took into account the fact that early rice, late rice, and in-season rice have different carbon emission coefficients. Consequently, the amounts of early rice, late rice, or in-season rice in different areas may have an effect on ACEs. The soil N_2_O emission coefficient is also influenced by the type of crop. The effective carbon emissions from livestock and poultry farming are mainly in the form of CH_4_ and N_2_O, and the emission coefficients vary with the species of livestock or poultry. All of the above were considered in this study, and the specific emission coefficients for rice, soil N_2_O, and livestock and poultry farming were obtained experimentally by other researchers.

Secondly, unlike in previous studies, this study attempted to consider agricultural straw burning as one of the sources of carbon emissions in order to make the calculated ACEs more accurate.

This study selected agricultural materials, rice cultivation, soil N_2_O emissions, livestock and poultry breeding, and straw burning as carbon sources to calculate the amount and the intensity of sources of ACEs in China’s 31 provinces from 1997 to 2016, and analyzed the temporal and spatial distribution of these ACEs. In this paper, when estimating the CH_4_ emissions from rice planting, the specific provinces and rice types were considered. When calculating ACEs from other sources, more specific information about the sources was considered. The carbon emission sources selected in this paper were based on previous research and were more comprehensive, and the results were therefore more accurate. According to calculation results, the following main conclusions can be drawn:

Firstly, between 1997 and 2016, the amount of ACEs in China increased, while the overall intensity of ACEs declined during the same period. The amount of ACEs from all source types, except rice planting, increased, and the relative contribution of the different source types to the total ACEs fluctuated. 

Secondly, the spatial distribution of the amount and intensity of ACEs varied greatly among provinces. The AACEs in traditional agricultural provinces were relatively high. On the whole, the IACEs of the provinces in Western China were relatively high, followed by the provinces of Central China, while the IACEs in the provinces in Eastern China were relatively low. The ACEs from different categories of carbon source showed different concentration trends and agglomeration forms.

Thirdly, both the amount and intensity of China’s ACEs showed obvious spatial correlation. Overall this correlation was high–high agglomeration; however, the spatial correlation gradually weakened during the study period. The locations of the spatial agglomerations of ACEs in each province changed from 1997 to 2016.

This study has some shortcomings. First, the selection of sources of ACEs can only reflect China’s actual ACEs to a certain extent. The carbon emission coefficients of more carbon emission sources need to be studied by researchers in relevant fields to allow ACEs to be measured in a more comprehensive way. Second, in this paper, straw burning was considered as one of the sources of carbon emissions. However, in recent years, China has made great efforts to promote environmental protection. Some local governments have now stopped farmers from burning straw, although straw has been burned for a long time. Considering the lag between the implementation of policy and its effect, this paper used straw burning as a carbon emissions source.

## Figures and Tables

**Table 1:** CH_4_ emission coefficients of different rice varieties in Chinese provinces (units: g/m^2^).

Province	ER	LR	IR	Province	ER	LR	IR	Province	ER	LR	IR
Beijing	0.0	0.0	13.23	Anhui	16.75	27.6	51.24	Sichuan	6.55	18.5	25.73
Tianjin	0.0	0.0	11.34	Fujian	7.74	52.6	43.47	Guizhou	5.1	21	22.05
Hebei	0.0	0.0	15.33	Jiangxi	15.47	45.8	65.42	Yunnan	2.38	7.6	7.25
Shaanxi	0.0	0.0	6.62	Shandong	0.00	0.00	21.00	Tibet	0	0	6.83
Inner Mongolia	0.0	0.0	8.93	Henan	0.00	0.00	17.85	Shaanxi	0	0	12.51
Liaoning	0.0	0.0	9.24	Hubei	17.51	39	58.17	Gansu	0	0	6.83
Jilin	0.0	0.0	5.57	Hunan	14.71	34.1	56.28	Qinghai	0	0	0.00
Heilongjiang	0.0	0.0	8.31	Guangdong	15.05	51.6	57.02	Ningxia	0	0	7.35
Shanghai	12.4	27.5	53.87	Guangxi	12.41	49.1	47.78	Xinjiang	0	0	10.50
Jiangsu	16.1	27.6	53.55	Hainan	13.43	49.4	52.29				
Zhejiang	14.4	34.5	57.96	Chongqing	6.55	18.5	25.73				

Note: ER = early rice, LR = late rice, IR = in-season rice.

**Table 2:** The CH_4_ and N_2_O emission coefficients of major livestock types (unit: kg/head/year).

Livestock	CH_4_ Emission Coefficient		N_2_O Emission Coefficient
	Enteric Fermentation	Manure Management	
Cow	68.000	16.00	1.00
Buffalo	55.000	2.00	1.34
Cattle	47.800	1.00	1.39
Mule	10.000	0.90	1.39
Camel	46.000	1.92	1.39
Donkey	10.000	0.90	1.39
Horse	18.000	1.64	1.39
Live pig	1.000	3.50	0.53
Sheep	5.000	0.15	0.33
Goat	5.000	0.17	0.03
Rabbit	0.254	0.08	0.02
Poultry	-	0.02	0.02

**Table 3:** Global Moran index for the amount and intensity of agricultural carbon emissions in China from 1997 to 2016.

Year	AACEs				IACEs			
	W_q_		W_d_		W_q_		W_d_	
	MI	PV	MI	PV	MI	PV	MI	PV
1997	0.184	0.036	0.062	0.007	0.299	0.000	0.044	0.000
1998	0.166	0.049	0.046	0.019	0.295	0.000	0.045	0.000
1999	0.167	0.048	0.040	0.028	0.295	0.000	0.049	0.000
2000	0.169	0.046	0.040	0.028	0.294	0.000	0.047	0.000
2001	0.175	0.041	0.038	0.032	0.291	0.000	0.043	0.000
2002	0.171	0.044	0.035	0.038	0.298	0.000	0.045	0.000
2003	0.154	0.059	0.025	0.064	0.297	0.000	0.046	0.000
2004	0.152	0.062	0.028	0.055	0.286	0.000	0.043	0.000
2005	0.140	0.073	0.022	0.075	0.293	0.000	0.046	0.000
2006	0.148	0.065	0.027	0.059	0.276	0.000	0.037	0.001
2007	0.151	0.062	0.029	0.052	0.222	0.001	0.027	0.001
2008	0.156	0.058	0.033	0.042	0.238	0.001	0.028	0.001
2009	0.154	0.059	0.035	0.038	0.246	0.001	0.032	0.001
2010	0.156	0.058	0.033	0.043	0.243	0.001	0.034	0.001
2011	0.150	0.063	0.031	0.047	0.251	0.001	0.035	0.001
2012	0.149	0.065	0.030	0.049	0.255	0.000	0.037	0.001
2013	0.145	0.069	0.029	0.053	0.259	0.000	0.038	0.001
2014	0.137	0.079	0.027	0.058	0.265	0.000	0.039	0.001
2015	0.140	0.075	0.028	0.057	0.267	0.000	0.037	0.001
2016	0.140	0.075	0.024	0.066	0.283	0.000	0.039	0.000

Note: AACEs: Amount of Agricultural Carbon Emissions; IACEs: Intensity of Agricultural Carbon Emissions; MI: Moran Index; PV: P-Value; W_q_: the first-order adjacent weight matrix; W_d_: the geographic distance matrix.
